# Biochemical changes in lumbar facet joint and disc degeneration by T2* mapping

**DOI:** 10.1186/s12891-024-07265-9

**Published:** 2024-03-20

**Authors:** Yi Ding, Liping Liu, Jiangyou Shi, Xiaodong Zhang, Rongchun Chen, Shuaishuai Xu

**Affiliations:** 1 Department of Spine Surgery, Ganzhou People’s Hospital, Ganzhou, Jiangxi 341000 China; 2 Department of Spine Surgery, Ganzhou Hospital, Jiangxi Medical College, Nanchang University, Ganzhou, Jiangxi 341000 China; 3 Department of Medical Imaging, Ganzhou People’s Hospital, Ganzhou, Jiangxi 341000 China; 4 Department of Nuclear Medicine, Shandong Provincial Hospital Affiliated to Shandong First Medical University, No.324 Jingwu Road, Jinan, Shandong 250021 China

**Keywords:** Magnetic resonance imaging, T2* mapping, Lumbar facet joint, Intervertebral disc

## Abstract

**Background:**

To investigate the biochemical changes in lumbar facet joint (LFJ) and intervertebral disc (IVD) with different degenerative grade by T2* mapping.

**Methods:**

Sixty-eight patients with low back pain (study group) and 20 volunteers (control group) underwent standard MRI protocols and axial T2* mapping. Morphological evaluation of LFJ and IVD were performed on T2-weighted imaging according to Weishaupt and Pfirrmann grading system, respectively. T2* values of LFJ and of AF (anterior annulus fibrosus), NP (nucleus pulposus), and PF (posterior annulus fibrosus) in IVD were measured. Kruskal-Wallis test and Wilcoxon rank-sum test were used to compare T2* values of subjects with different degenerative grade.

**Results:**

The mean T2* value of grade 0 LFJ (21.68[17.77,26.13]) was higher than those of grade I (18.42[15.68,21.8], *p* < 0.001), grade II (18.98[15.56,22.76], *p* = 0.011) and grade III (18.38[16.05,25.07], *p* = 0.575) LFJ in study group, and a moderate correlation was observed between T2* value and LFJ grade (rho=-0.304, *p* < 0.001) in control group. In the analysis of IVD, a moderate correlation was observed between AF T2* value and IVD grade (rho=-0.323, *p* < 0.001), and between NP T2* value and IVD grade (rho=-0.328, *p* < 0.001), while no significant difference was observed between the T2* values of PF in IVD of different grade in study group.

**Conclusions:**

Downward trend of T2* values can be found in LFJ, AF and NP as the degenerative grade rised. But in elderly patients with low back pain, no change trend was found in LFJ due to increased fluid accumulation in the joint space.

**Supplementary Information:**

The online version contains supplementary material available at 10.1186/s12891-024-07265-9.

## Background

Low back pain is a musculoskeletal disorder that affects up to 60–80% of the population at some point during their lifetime, resulting in considerable negative impacts on quality of life and social economy [1–4]. Both lumbar facet joint (LFJ) osteoarthritis (OA) and intervertebral disc (IVD) degeneration are regarded as common cause of low back pain [5–8]. Thus, it is critical to accurately evaluate the status of LFJ and IVD at different grade of disease process in a reproducible manner.

MRI is an useful imaging modality for evaluating the morphological changes of lumbar three-joint complex including LFJ and IVD in clinical practice. The signal characteristics on T2-weighted images reflect changes caused by aging or degeneration [9, 10]. However, conventional MRI protocols are difficult to detect early grade of degenerative changes, and the reproducibility of grading systems based on morphologic changes are non-satisfactory [11]. With the development of MRI protocols over the past two decades, researchers have recently shown increased interests in biochemical quantitative imaging techniques, such as T2, T2*, and T1ρ mapping [12–16]. Among which T2* value reflects the “true” transverse relaxation time and decreases with an increase in cartilage degeneration [17, 18]. Several studies have demonstrated that T2* mapping is a reliable and valid diagnostic method in biochemical cartilage imaging that can be implemented into clinical MR protocol [19, 20], and has been proved to be effective to detect degenerative changes in lumbar facet joint and intervertebral disc [12, 21, 22].

Thus, the aim of this study was to investigate the biochemical changes in LFJ and IVD with different degenerative grade by T2* mapping.

## Methods

This retrospective study had received the institutional review board approval, and was performed with waiver of informed consent.

### Patient population

Patients suffering from low back pain originating from lumbar spine who had undergone standard MRI protocols and axial T2* mapping between January 1, 2020 to June 1, 2023 were included in this study. Exclusion criteria: (1) patients with lumbar tuberculosis, lumbar IVD infection, severe lumbar hypoplasia, blood disease involving the lumbar spine, lumbar spine tumor, or concomitant skeletal-rheumatoid disease at the time of MRI examination; (2) MRI revealed abnormal signal in paraspinal muscle or sacroiliac joint lesions. Patient information was anonymized and de-identified prior to analysis.

### Image acquisition and analysis

Patients were scanned using a 3.0 T MRI unit (Tim Trio, Siemens Medical Solutions, Erlangen, Germany) with a dedicated 8-channel spine coil. Axial T2* mapping used the following parameters: fast spin echo, repetition time 575 ms, echo time 4.2, 11.3, 18.5, 25.6, 32.7 ms, field of view 160 × 160 mm, voxel size 0.4 × 0.4 × 4.0 mm, interslice gap 0.3 mm, number of slices 15, examination time 3 min 41 s.

Morphological evaluation of LFJ OA was performed on T2-weighted imaging (T2WI) according to Weishaupt grading system [9]. To exclude the subjective factor and facilitate further analysis, IVD degeneration was evaluated according to modified Pfirrmann grading system [10]: grade I, Pfirrmann 1; grade II, Pfirrmann 2; grade III, Pfirrmann 3 to 4; grade IV, Pfirrmann 5 to 8 (Table 1). Image analysis was performed by one radiologist and one spine surgeon to evaluate inter-observer reliability.

**Table 1:** Pfirrmann grading system of IVD on T2WI

Grade	Signal intensity of nucleus pulposus and inner fibrosus	Signal difference between rear, inner and outer fibrosis	IVD height
1	Uniform high signal (nearly equal to CSF)	Obvious	Normal
2	High signal (higher than presacral fat, but lower than CSF)	Obvious	Normal
3	High signal (cracks in the nucleus pulposus observed)	Obvious	Normal
4	High signal (lower than presacral fat)	Not obvious	Normal
5	Low signal (equal to outer fibrosus)	Not obvious	Normal
6	Low signal	Not obvious	Reduce < 30%
7	Low signal	Not obvious	Reduce 30–60%
8	Low signal	Not obvious	Reduce > 60%

IVD, intervertebral disc; CSF, cerebrospinal fluid

**Fig. 1:**
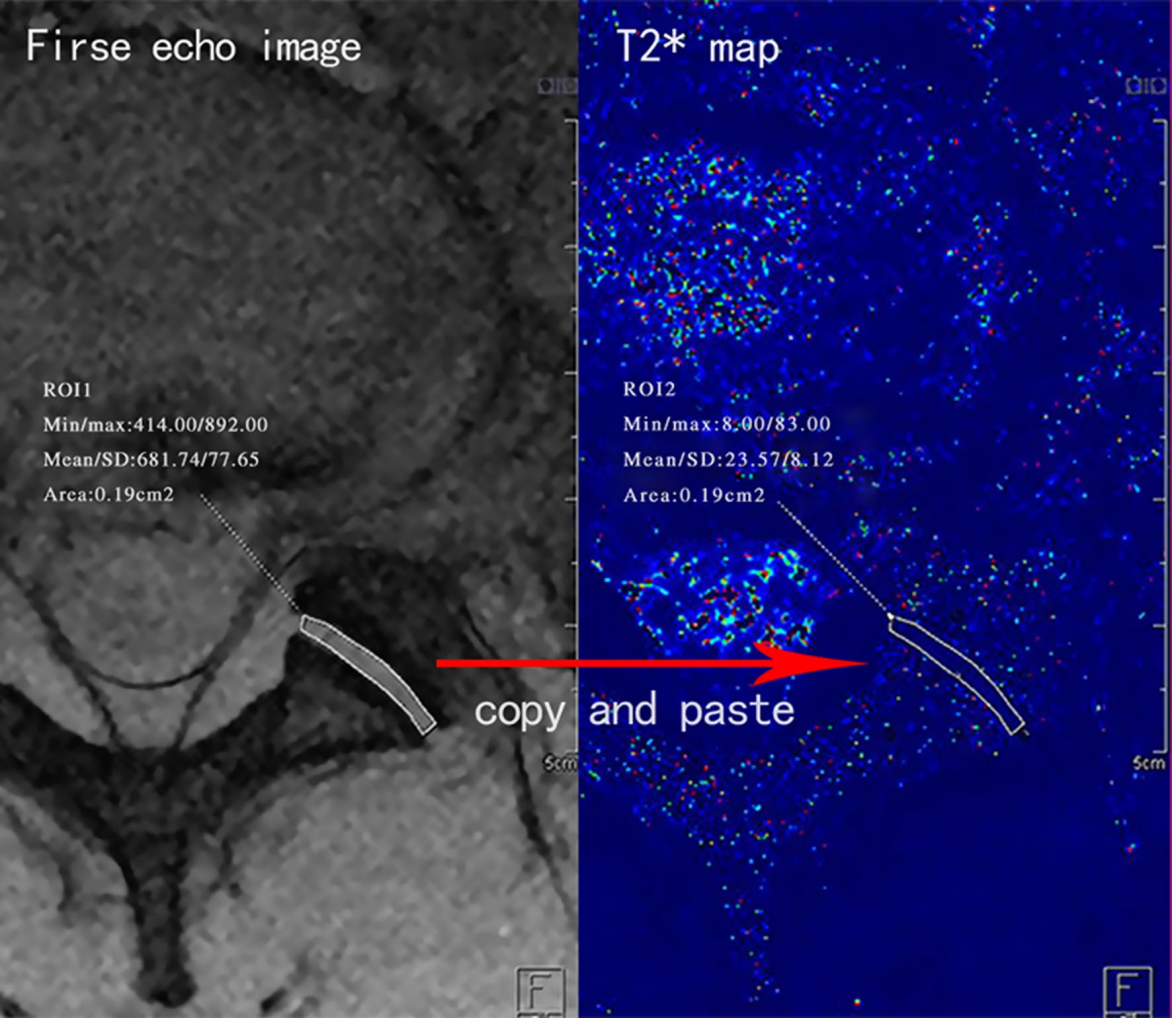
Measurement of T2* value of lumbar facet joint. Region of interest was primarily delineated on first echo anatomical image (left) and copied to the corresponding T2* mapping image (right)

**Fig. 2:**
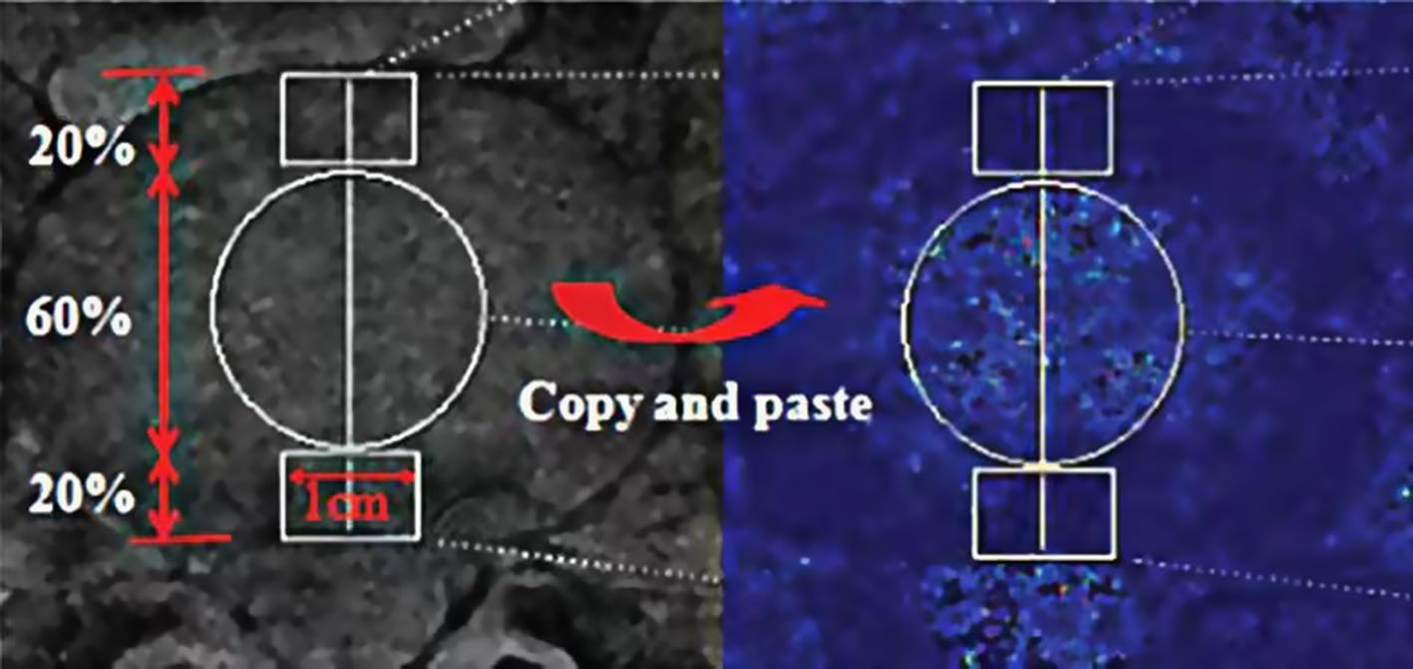
Measurement of T2* value of lumbar intervertebral disc. A length was drawn from the anterior edge to the posterior edge and divided by 2:6:2 on first echo anatomical image (left). Regions of interest of anterior AF, posterior AF, and NP were manually delineated, respectively, and copied to the corresponding T2* mapping image (right)

For the measurement of T2* value of LFJ and IVD, region of interest (ROI) was primarily delineated on first echo anatomical image and copied to the corresponding T2* mapping image (Figs. 1 and 2). For IVD, a length was drawn from the anterior edge to the posterior edge and divided by 2:6:2 on first echo anatomical image. ROIs of AF (anterior annulus fibrosus), NP (nucleus pulposus), and PF (posterior annulus fibrosus) were manually delineated, respectively (Fig. 2).

### Statistical analysis

Shapiro-Wilk test was used to determine whether the continuous variables accord with normal distribution. Continuous variables were presented as means and SD (normal distribution), or as medians and quartiles (non-normal distribution). Age and gender were compared between the study and control subjects using student t test and Chi-squared test, respectively. Inter-observer reliability for continuous and categorical variables were respectively evaluated using intraclass correlation coefficient (ICC) and Kappa value, interpreted as follows: 0-0.3, weak agreement; 0.3–0.5, moderate agreement; 0.5–0.7, substantial agreement; 0.7–1.0, almost perfect agreement. For evaluating the correlation between continuous variables and ordered categorical variables, one way ANOVA (normal distribution with equal variance) or Kruskal-Wallis test (non-normal distribution) and Spearman rank test were used. For evaluating the correlation between continuous variables and categorical variables with only two levels, student t test or Wilcoxon rank-sum test was used.

All reported p values were two-sided. A p value of < 0.05 was considered statistically significant. All statistical analyses were performed using R-4.2.3 (https://www.r-project.org).

## Results

### Patient clinical features

We included 327 lumbar 3-joint complexes from 68 patients (36 males, 32 females; median age, 47.9 ± 13.7 years; range, 34–83 years) in the study group, and 99 lumbar 3-joint complexes from 20 volunteers (11 males, 9 females; mean age, 24.2 ± 1.3 years; range, 22–27 years) in the control group. The mean age of patients in study group was significantly higher (*p* < 0.001) than that in control group, and no significant difference was observed in gender ratio between the two groups (*p* = 0.871).

### T2* values of LFJ

Weishaupt grading results of LFJ were summarized in Table 2. The prevalence of advanced LFJ degeneration (Weishaupt grade II and III) in study group was significantly higher (*p* < 0.001) than that in control group. Inter-observer reliability for the measurement of of LFJ T2* value was almost perfect (ICC = 0.95, [0.943–0.957]).

**Table 2:** Weishaupt grading results of LFJ

	0 (%)	I (%)	II (%)	III (%)
Study	68 (10.4)	412 (63.0)	130 (19.9)	44 (6.7)
Control	27 (13.6)	144 (72.7)	25 (12.6)	2 (1.0)
Total	95 (11.2)	556 (65.3)	155 (18.2)	46 (5.4)

LFJ, lumbar facet joint

**Fig. 3:**
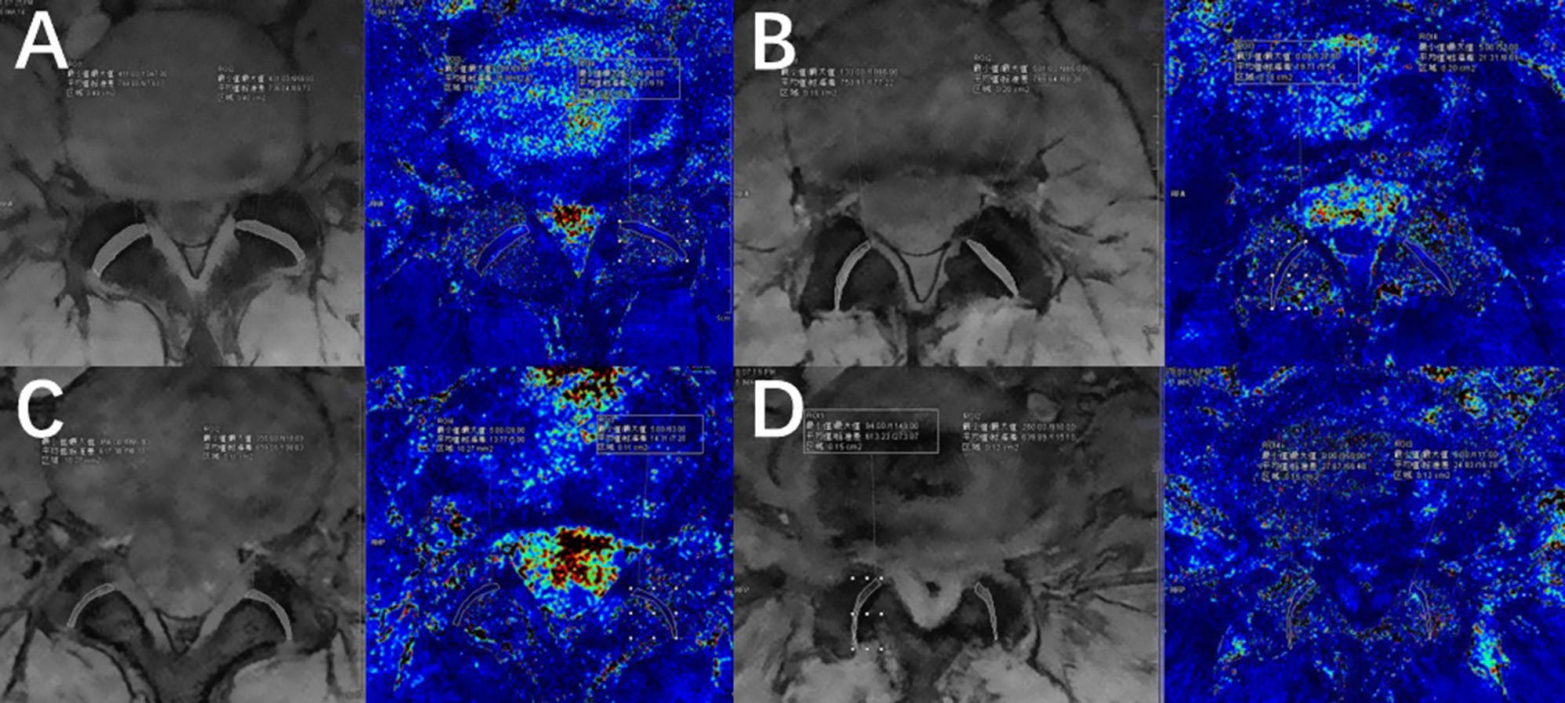
A 52-year-old male patient with low back pain showed bilateral grade 0 lumbar facet joint (LFJ) and grade I intervertebral disc (IVD) at first echo anatomical image and T2* mapping image (**A**). A 39-year-old male patient with low back pain showed bilateral grade I LFJ and grade II IVD at first echo anatomical image and T2* mapping image (**B**). A 38-year-old female patient with low back pain showed left grade II LFJ and right grade I LFJ, and grade II IVD at first echo anatomical image and T2* mapping image (**C**). A 65-year-old female patient with low back pain showed left grade III LFJ and right grade II LFJ, and grade IV IVD at first echo anatomical image and T2* mapping image (**D**)

In the study group, the mean T2* value of grade 0 LFJ was significantly higher than those of grade I (*p* < 0.001) and grade II LFJ (*p* = 0.011), and was higher than grade III LFJ but not reaching a significant difference (*p* = 0.575). No significant difference was observed between the mean T2* values of grade I and II LFJ (*p* = 0.764), grade I and III LFJ (*p* = 0.749), grade II and III LFJ (*p* = 0.764). No significant correlation was observed between T2* value and LFJ grade (rho=-0.06, *p* = 0.1254) (Table 3; Fig. 3) (Supplementary Material 1).

**Table 3:** T2* values of LFJ in study and control groups

	0	I	II	III	p value	rho (p value)
Study (ms)	21.68(17.77,26.13)	18.42(15.68,21.8)	18.98(15.56,22.76)	18.38(16.05,25.07)	0.0014	-0.06 (0.1254)
Control (ms)	20.15(17.83,24.21)	17.2(14.84,19.97)	15.41(12.87,17.51)	14.91(14.81,15.0)	0.0004	-0.304 (< 0.001)
p value	0.4779	0.0047	0.0006	0.1165		

LFJ, lumbar facet joint

In the control group, the mean T2* value of grade 0 LFJ was significantly higher than those of grade I (*p* = 0.013) and grade II LFJ (*p* = 0.001). The mean T2* value of grade I LFJ was significantly higher than that of grade II (*p* = 0.046). Due to the small sample size of grade III LFJ of only two, no significant difference was observed between the mean T2* values of grade 0 and III LFJ (*p* = 0.233), grade I and III LFJ (*p* = 0.526), grade II and III LFJ (*p* = 0.817). A moderate correlation was observed between T2* value and LFJ grade (rho=-0.304, *p* < 0.001) (Table 3).

Interestingly, the mean T2* values of grade I (*p* = 0.005) and II (*p* < 0.001) LFJ in study group were significantly higher than those in control group. Due to the small sample size of grade III LFJ of only two in control group, the mean T2* values of grade III LFJ in study group was higher than that in control group but not reaching a significant difference (*p* = 0.575) (Table 3).

### T2* values of IVD

Modified Pfirrmann grading results of IVD in study and control groups were summarized in Table 4. The prevalence of advanced IVD degeneration (modified Pfirrmann grade III and IV) in study group was significantly higher (*p* < 0.001) than that in control group. Inter-observer reliability for the measurement of AF, NP, and PF T2* values was almost perfect with ICC of 0.953 (0.943–0.962), 0.971 (0.965, 0.977), and 0.896 (0.79, 0.939), respectively.

**Table 4:** Modified Pfirrmann grading results of IVD

	I (%)	II (%)	III (%)	IV (%)
Study	90 (27.5)	47 (14.4)	115 (35.2)	75 (22.9)
Control	50 (50.5)	46 (46.5)	3 (3.0)	0 (0)
Total	140 (32.9)	93 (21.8)	118 (27.7)	75 (17.6)

IVD, intervertebral disc

In the study group, the mean T2* value of grade I AF was significantly higher than those of grade III (*p* < 0.001) and grade IV AF (*p* < 0.001), and was higher than grade II AF but not reaching a significant difference (*p* = 0.235). The mean T2* value of grade II AF was significantly higher than that of grade IV (*p* < 0.001), and was higher than grade III AF but not reaching a significant difference (*p* = 0.235). A moderate correlation was observed between AF T2* value and IVD grade (rho=-0.323, *p* < 0.001). The mean T2* value of grade I NP was significantly higher than that of grade IV NP (*p* < 0.001), and was higher than grade III NP but not reaching a significant difference (*p* = 0.16). The mean T2* value of grade II NP was significantly higher than that of grade IV NP (*p* < 0.001), and was higher than grade III NP but not reaching a significant difference (*p* = 0.16). The mean T2* value of grade III NP was significantly higher than that of grade IV NP (*p* < 0.001). A moderate correlation was observed between NP T2* value and IVD grade (rho=-0.328, *p* < 0.001). No significant difference was observed between the T2* values of PF in IVD of different grade (*p* = 0.1294), and no significant correlation was observed between PF T2* value and IVD grade (rho=-0.053, *p* = 0.3356) (Table 5).

**Table 5:** T2* values of IVD with modified Pfirrmann grade of IVD

		I	II	III	IV	p value	rho (p value)
AF	Study (ms)	52.14(36.7,81.41)	44.33(31.17,69.74)	37.68(26.86,50.36)	32.94(25.34,42.02)	< 0.001	-0.323 (< 0.001)
	Control (ms)	56.96(36.61,63.85)	46.88(37.94,82.73)	40.99(36.84,62.68)	/	0.8127	0.026 (0.7994)
	p value	0.8534	0.0917	0.5214	/		
NP	Study (ms)	61.41(43.49,79.45)	63.68(42.17,89.17)	51.48(38.83,71.19)	37.86(32.23,46.85)	< 0.001	-0.328 (< 0.001)
	Control (ms)	87.24(66.55,114.03)	96.8(58.26,116.85)	101.28(77.56,104.14)	/	0.8542	0.049 (0.6301)
	p value	< 0.001	< 0.001	0.007	/		
PF	Study (ms)	24.63(20.48,831.34)	28.36(22.15,34.85)	26.31(20.19,35.31)	23.07(19.39,31.84)	0.1294	-0.053 (0.3356)
	Control (ms)	28.53(23.35,36.78)	32.49(22.84,45.39)	21.82(21.67,31.73)	/	0.4204	0.062 (0.5416)
	p value	0.0032	0.0256	0.8392	/		

IVD, intervertebral disc; AF, anterior annulus fibrosus; NP, nucleus pulposus; PF, posterior annulus fibrosus

In the control group, no significant difference was observed between the T2* values of AF (*p* = 0.813), NP (*p* = 0.854) and PF (*p* = 0.42) in IVD of different grade due to the small sample size of grade III IVD of only three and that of grade IV IVD of zero. And no significant correlation was observed between T2* values and IVD grade (Table 5).

Interestingly, the mean T2* values of grade I (*p* < 0.001) and II (*p* < 0.001) NP, and of grade I (*p* = 0.003) and II (*p* < 0.026) PF in control group were significantly higher than those in study group. As the sample size of grade III and IV IVD was too small in the control group, the relevant analysis results were ignored (Table 5).

## Discussion

Both T2 and T2* values are sensitive to water content and interactions between water molecules and collagen fibers, and high values always indicate high water content and superior water molecule mobility [23]. Different from T2 relaxation, T2* relaxation is unique for gradient-echo sequences. It is a combination of “true” T2 relaxation and relaxation caused by magnetic field inhomogeneity. Thus, T2* value is shorter than T2 value, and their relationship can be expressed by the following equation, where γ is the gyromagnetic ratio: 1/T2* = 1/T2 + γ ΔB_inhom_ ,or 1/T2* = 1/T2 + 1/T2′, where 1/T2′ = γ ΔB_inhom_, and ΔB_inhom_ is the magnetic field inhomogeneity across a voxel [24]. T2* mapping provides information about the spatial macromolecule architecture and its interaction with water mobility, and T2* value has been proposed as a robust biomarker of cartilage degeneration not only in the spine but also in other joints including hip, knee, and ankle [19, 22–24].

In the study group, we found a downward trend of T2* value of NP as the degenerative grade of IVD rised, which is consistent with previous reports [14, 22, 25]. Interestingly, a downward trend of T2* value of AF was also observed. As the gelatinous structure of NP consists mostly of water with a low yield of collagenous material, it is easily to understand that water content in NP linearly decreased as the IVD degenerated progressively, resulting in a downward trend of T2* value. But the annulus fibrosus is mainly composed of fibrocartilage containing a fibrous structure and low water content. A possible explanation may be that the distribution of water content in annulus fibrosus is not homogeneous, with richer water content in AF than that in PF. In the control group, due to the small sample size of grade III IVD of only three and that of grade IV IVD of zero, the change trend of T2* value of IVD of different degenerative grades can not be evaluated.

In the analysis of LFJ, the mean T2* value of grade 0 LFJ was higher than those of grade I, grade II and grade III LFJ, and no significant difference was observed between the mean T2* values of grade I, II and III LFJ in study group. Thus, no change trend was found in the T2* value of LFJ. In contrast, a downward trend of T2* value was observed as the degenerative grade of LFJ rised. We hypothesized that in elderly patients with low back pain of the study group, fluid accumulation in the joint space increased as the LFJ degenerated progressively. Which would offset the degeneration of articular cartilage, finally resulting in increased water content in LFJ. The significantly higher mean T2* values of grade I and II LFJ in study group than those in control group also support our hypothesis.

Another finding was that the mean T2* values of grade I and II NP, and of grade I and II PF in control group were significantly higher than those in study group. Due to the significantly higher mean age of patients in study group than that in control group, age-related degeneration may be a possible explanation. In contrast, the mean T2* values of grade I and II LFJ in study group were significantly higher than those in control group. The increased fluid accumulation in the degenerated joint space described above may be a possible reason.

The subjects we collected in this study were outpatient patients referred for low back pain. Chronic low back pain related to LFJ and IVD degeneration is often resulted from the degeneration, osteoarthritis, and effusion of joints, which are closely related the damage and degeneration of joint cartilage. Although LFJ and IVD degeneration are regarded as common causes of low back pain, it also can be caused by many other reasons [5, 7, 8]. Thus, accurately determining the direct cause of low back pain play an important role in the planning of treatment [5, 7, 8]. As the level of T2* value can reflect the degree of LFJ and IVD degeneration, it may be useful to help clinicians determine whether the pain comes from LFJ or IVD.

Several limitations in the current study. First, there was no histopathological assessment of LFJ and IVD degeneration. This is difficult to achieve in humans, and further experimental research on animals is needed. Secondly, the imaging time of all participants was uncertain, ignoring the diurnal variation of facet joints and discs as confirmed by prior studies. Thirdly, the number of participants, especially of volunteers was relatively small. Further investigation is necessary to assess whether our results would be obtained with a larger number of participants.

## Conclusions

Downward trend of T2* values can be found in LFJ, AF and NP as the degenerative grade rised. But in elderly patients with low back pain, no change trend was found in LFJ due to increased fluid accumulation in the joint space.

### Electronic supplementary material

Below is the link to the electronic supplementary material.

Supplementary Material 1

## Data Availability

Data is provided within the supplementary information files.
